# Relationship between first-aid literacy, coping style, family functioning and mental health of caregivers of children with asthma

**DOI:** 10.1186/s12912-026-04829-z

**Published:** 2026-05-31

**Authors:** Mingjing Fu, Xiujie Sun, Yaoyao Zhang, Kun Chi, Shanshan Huan, Zihan Gao, Huan Han, Wenjing Wang, Xiangdi Dong

**Affiliations:** Qingdao Hospital, University of Health and Rehabilitation Sciences (Qingdao Municipal Hospital), 5 Donghai Middle Road, Shinan District, Qingdao City, Shandong Province 266071 China

**Keywords:** Asthma, First-aid literacy, Coping style, Family function, Mental health

## Abstract

**Background:**

The rising prevalence of childhood bronchial asthma imposes a substantial economic burden and threatens the mental health of affected families, worldwide. Although multiple factors influence caregivers’ psychological wellbeing, systematic investigations in this area are still lacking. Hence, this study aimed to explore the relationship between first-aid literacy, coping style, family functioning, and the mental health of caregivers of children with asthma.

**Methods:**

This cross-sectional descriptive study included 337 children with asthma and their parents. Survey questions assessed demographic and disease related data and caregivers’ first-aid literacy, coping style, family functioning, and the mental health using validated tools. Descriptive statistics and Pearson’s correlation analysis were used to analyse the data. The maximum likelihood method was used to fit and modify the relational model.

**Results:**

First-aid literacy and coping styles were inversely correlated with caregivers’ mental health, whereas family functioning was positively associated. Additionally, first-aid literacy was positively correlated with coping style and negatively correlated with family functioning (*P* < 0.01). First-aid literacy not only directly affected the caregivers’ mental health (*β* = 0.334), but also indirectly impacted it through the mediation of coping style and family functioning (*β* = 0.453). Coping style and family functioning played important mediating roles in the relationship between first-aid literacy and caregivers’ mental health.

**Conclusion:**

First-aid literacy played an important role in improving caregivers’ mental health. Therefore, healthcare professionals, particularly nurses who have frequent contact with caregivers, should pay attention to first-aid literacy and take effective measures to improve the mental well-being of caregivers’ of children with asthma.

**Clinical trial number:**

Not applicable.

**Supplementary Information:**

The online version contains supplementary material available at 10.1186/s12912-026-04829-z.

## Significance

What is already known about this topic?

What does this article add to our knowledge?

How does this study impact current management guidelines?

Caregivers of children with asthma experience mental health challenges owing to environmental factors and stressors. Psychological well-being is also influenced by coping style and family functioning.

This study integrated the Pearlin’s stress model to elucidate the pathways through which environmental factors, stressors, and care-regulating factors affect the caregivers’ mental health. It further reveals that first-aid literacy directly affects mental health, and influences coping mechanisms and family dynamics. These findings highlight the multifaceted nature of factors influencing caregivers’ well-being.

This study provides a theoretical framework to guide the development of more comprehensive mental health education programs for caregivers. It suggests incorporating first-aid literacy, coping skills, and family functions into interventions, while considering broader environmental stressors. This holistic approach can enhance the current guidelines for supporting caregivers of children with asthma.

**Supplementary Information:**

The online version contains supplementary material available at 10.1186/s12912-026-04829-z.

## Introduction

The third phase of the International Study of Asthma and Allergies in Childhood reported that 13.7% of the children worldwide have asthma [1]. A recent epidemiological survey found that the incidence of childhood asthma in China has reached 3.02%, an increase of 50.6% compared with a previous survey [2, 3]. Paediatric asthma has a higher incidence of disease recurrence than other chronic diseases, further increasing the existing caregiver burden. In China, there are significant regional variations in prevalence of childhood asthma across the country owing to differences in air pollution, climate, medical resources, and health literacy. Qingdao, an eastern coastal city in China that has undergone rapid industrialisation and urbanisation, has shown 3,69% of bronchial asthma among children [4], which is higher than the national average, placing a great burden on caregivers. Recurrent asthma attacks and long-term treatment demands create significant care burden and psychological stress for caregivers [5]. Caregivers play a key role in the management of asthma in children and are responsible for long-term drug monitoring and symptom management, in addition to meeting the daily needs of family life such as cooking, housework, and tutoring. However, no studies have specifically explored the mental health status of caregivers of children with asthma in Qingdao, or the factors influencing it (such as first-aid knowledge, coping styles, and family function). Understanding these issues in this urban Chinese context can generate evidence relevant to other rapidly developing regions facing similar environmental and healthcare challenges, thereby extending the applicability of our findings beyond Qingdao.

According to Pearlin’s caregiver stress model [6], caregiver stress is influenced by many factors including environmental factors, stressors, regulatory factors, and caregiver outcomes. Environmental factors include caregiver characteristics (e.g. first-aid literacy, age, education, and income). Stressors are divided into primary and secondary stressors; primary stressors refer to child-related factors (e.g. the child’s age, sex, disease stage, and frequency of asthma attacks) whereas secondary stressors refer to the caregiver’s family environmental factors (e.g. monthly family income, family functioning, and internal family relationships). Regulatory factors refer to coping styles, and caregiver outcomes include mental and physical health. First-aid literacy is defined as individuals’ cognition and ability in providing first aid [7].

Studies have shown that the incidence of anxiety and depression among caregivers of children with asthma is 23.56%, higher than that for caregivers of children with other conditions [8]. Recent studies have shown that caregivers’ anxiety can affect their ability to care for children and manage their disease, which is not conducive to asthma recovery [9]. Since there is substantial evidence that caregivers can induce both negative emotions such as anxiety and depression, and positive emotions such as self-affirmation, in the process of taking care-giving, positive emotions can enable caregivers to cope better with emergencies and manage children’s diseases [9]. Moreover, caregivers’ knowledge of asthma disease management and first aid positively impact children’s disease recovery and mental health [10].

Along with first-aid literacy, coping styles are also closely associated with caregivers’ mental health. Evidence suggests that a positive coping style is conducive to caregivers’ mental health [11]. Additionally, face-to-face interviews with caregivers of children with asthma revealed a correlation between family functioning and caregiver mental health [12]. Family functioning can be defined as a comprehensive evaluation of the operational status of the family system and the relationships among family members [13]. Furthermore, there is theoretical and empirical basis for positing that first-aid literacy influences family functioning. Caregivers with higher first-aid literacy can better manage acute asthma exacerbations calmly and effectively, which reduces the frequency and intensity of asthma-related crises. Fewer crises reduce family disruptions, role strain, and conflict, thereby preserving or improving family functioning. This reasoning is consistent with broader health literacy research. For example, Saleem et al. found that higher health literacy was significantly associated with better social and family support [14]. A systematic review by Tzavara et al. concluded that health literacy was a key explanatory factor in the relationship between family cohesion and health outcomes [15]. In the context of chronic paediatric diseases, Mackey et al. reported that parental health literacy directly influenced disease self-management capacity, reducing emergency events and hospitalisation factors that protect family routines and reduce conflict [16]. Similarly, higher health literacy among parents of chronically ill children was also shown to be associated with greater self-efficacy and fewer caregiving disruptions, thereby preserving family functioning [17]. Higher caregiver health literacy was also found to be correlated with fewer caregiving challenges, indirectly supporting the role of literacy in maintaining harmonious family functioning [18]. Collectively, these studies provide a strong rationale for the hypothesis that first-aid literacy is an antecedent of family functioning in paediatric asthma. Moreover, the experience of caring for children with chronic disease can itself affect family functioning [19]. The prolonged and recurrent nature of asthma care often requires caregivers to devote substantial time and energy to disease management, which may lead to reduced attention being paid to other family members, disrupted family routines, and increased family conflicts. Thus, family functioning may deteriorate because of caregiving for children with asthma.

Although separate associations between coping style, family functioning, and caregivers’ mental health have been reported in some studies, few studies have simultaneously examined how first-aid literacy, coping style, and family functioning collectively influence the mental health of caregivers of children with asthma, particularly within a structural equation model (SEM) based on Pearlin’s caregiver stress framework. This study is the first to do so as it aimed to explore the relationship between first-aid literacy, coping style, family function, and the mental health of caregivers of children with asthma, and proposes intervention measures to improve these caregivers’ mental health.

Building on Pearlin’s theoretical framework, we hypothesised that:

1. *First-aid literacy is strongly associated with caregivers’ mental health.*
2. *Coping style and family functioning mediates the relationship between first-aid literacy and mental health.*

## Materials and methods

### Study design and setting

This study was designed as a cross-sectional survey study that used convenience sampling to select the research participants because caregivers of children with asthma are a dispersed population, and recruiting all eligible caregivers attending the study hospital during the study period was the most feasible approach given the time and resource constraints.

Based on this equation, $N = {{{Z^2}_{\alpha /2}\left( {1{\rm{ - }}P} \right)} \over {{\varepsilon ^2}P}}$, derived from a previous study [20], where *P* = 0.24, α = 0.05, ε = 0.2, and Z α/2 = 1.96, the minimum sample size was estimated as 304. After adding 5% invalid responses, the target was 320 participants. The study was conducted between August 2020 and January 2021.

### Participants

The participants were 337 children with asthma admitted to the children’s internal medicine clinic of a third-class A hospital in Qingdao, and their caregivers, who provided valid responses. Inclusion criteria for children were: (a) age ranging from 4 to 11 years; (b) asthma diagnosis according to the Diagnostic Criteria of the 2020 Guidelines for the Diagnosis, Prevention, and Treatment of Bronchial Asthma [21]; (c) the course of the disease > 3 months; (d) no family history of psychosis. Inclusion criteria for caregivers were: (a) relatives of the respective participant children, such as parents (b) should be taking care of the children for ≥ 3 months (this threshold is methodologically justified as conditions lasting ≥ 3 months are generally classified as “chronic” and distinguished from acute episodes [22], and this has been widely adopted in caregiver research to ensure sufficient chronic caregiving experience while minimising recall bias [23]); (c) with adequate reading and cognition ability. Children with asthma complicated with other chronic diseases (diabetes, epilepsy, migraine, tumour), b) children with acute attacks, and c) caregivers with mental or other serious illnesses who were unable to complete the questionnaire independently were excluded from the study.

Participating children’s average age was 7.28 ± 1.95 years, and the sample included 226 boys and 111 girls. Fifty-two children were in clinical remission and 285 were in chronic remission. Children’s caregivers (*n* = 337) had an average age of 36.04 ± 3.15 years, and included 103 men and 234 women.

### Measurements

#### The General Data Questionnaire

Based on previous relevant literature, the researcher designed a General Data Questionnaire to collect basic information on participants and disease. The children’s section included general information, such as age, gender, single child status, as well as disease information such as disease years and symptom control. Caregivers’ information included their age, gender, relationship with the child, working status, occupation, daily care time, monthly family income, etc. This questionnaire consisted of 27 items: 12 items pertaining to children with asthma and 13 items pertaining to their caregivers, with an additional 2 items completed by the researchers to assess asthma disease stage and disease control level, as detailed in the Supplementary Materials.

#### First-aid literacy

First-aid literacy was assessed using the safety and first-aid contents of the Health Literacy Questionnaire for Chinese residents compiled by the China Health Education Center [24]. It comprises 10 single-choice, short, and judgement-based questions on basic first-aid knowledge and skills such as on-site self-rescue and mutual rescue, and emergency treatment knowledge. The score ranged between 0 and 14 points, with a score ≥ 11 indicating adequate first-aid literacy [25]. Following Stadler et al.’s work [26], the first-aid literacy scale was conceptualised as a formative construct. Consequently, traditional internal consistency indicators were not applicable. The scale’s validity was supported by its origin from a national health-literacy survey [27], and its utility was further confirmed through significant SEM pathways.

#### Coping style

Coping style was assessed via the Coping Health Inventory for Parents (CHIP), originally developed by Hamilton et al. [28]in the U.S, and later translated into Chinese by Yang [29]. This scale includes two dimensions: the frequency of the parental positive response method and parents’ positive coping methods work. Caregivers choose the frequency of using the 45 positive coping styles and the effect of using the corresponding positive coping styles on maintaining a normal family life. The higher the score on the scale, the higher the frequency of the parental positive response method and parents’ positive coping methods work. This scale had a Cronbach’s α of 0.89 in a prior study [30], and 0.785 in the present study.

#### Family functioning

Family functioning was assessed via the Family Assessment Device (FAD), originally compiled by Epstein according to McMaster’s family function model theory [31], and the Chinese version was later revised by Wang [32]. This scale is used to collect information from all aspects of the family and is a simple and effective for evaluating problems in the family [33]. This study used the General Function sub-scale, which is a self-rating scale of 12 items in total. Higher scores on this indicate poorer (unhealthy) family functioning. This scale had a Cronbach’s α of 0.78 in a prior study [34], and 0.725 in the present study.

#### Mental health

Participants’ mental health was assessed using The General Mental Health Questionnaire (GHQ-20), a 20-item derivative of the original Goldberg General Health Questionnaire originally compiled by Goldberg [35], and later translated and revised by Chinese scholars to form the Chinese version of the General Mental Health Questionnaire [36]. This scale comprises 20 items that assess self-affirmation, depression, and anxiety. Questions 7 and 10 are reversed scored. The higher the score on each sub-dimension, the higher is the degree of that dimension. Higher total scores indicate poorer mental health (greater psychological distress). The Chinese version of the GHQ-20 has been validated for assessing anxiety and depression tendencies (reported reliability 0.938 [37]; present study Cronbach’s α = 0.816), demonstrating acceptable internal consistency and construct validity. The Chinese GHQ-20 has been validated for assessing anxiety and depression tendencies in caregivers of children with asthma [38].

### Ethical considerations

All procedures performed in this study involving human participants were in accordance with the ethical standards of the institutional research committee and with the 1964 Helsinki Declaration and its later amendments. This study was approved by the Ethics Committee of the Affiliated Hospital of Qingdao University (approval no. QYFYWZLL25906). Written informed consent was obtained from all participants before the survey. Participation was voluntary, and data confidentiality was ensured.

### Data collection

Before the investigation, families with asthma were informed of the study aim, voluntary participation, and the significance of the study. Only the caregivers completed the questionnaires at the outpatient clinic of the hospital. After completion, the questionnaires were immediately retrieved and checked to identify any missing sections. A total of 350 families were enrolled in the study, and 13 (3.7%) were excluded owing to incomplete data. Finally, 337 families (96.3%) provided complete data on the variables assessed in this study.

### Data analysis

All statistical analyses were performed using SPSS 25.0, and AMOS 24.0. Descriptive statistics of the study population were calculated to estimate the means (Ms) and standard deviations (SDs) for continuous variables and percentages for categorical variables. Pearson’s correlation analysis was used to analyse the correlations among first-aid literacy, coping style, family function, and mental health. Amos software (version 24.0) was used to construct a model of first-aid literacy, coping style, family function, and mental health of caregivers. Additionally, the total and indirect effects were calculated. Chi-squared test statistics, comparative fit index (CFI), root mean square error of approximation (RMSEA), and standardised root mean square residual (SRMR) were reported. Based on literature review, the following cut-off scores were selected for good fit: CFI ≥ 0.95, RMSEA ≤ 0.06 and SRMR ≤ 0.08 [39]. A value of *P* < 0.05 was accepted as statistically significant.

## Results

### Participant characteristics

These demographic statistics are detailed in Table 1.

**Table 1:** Demographic data of asthmatic children and their caregivers (*N* = 337)

Variable		*N* (%)
Disease staging	Chronic duration	285 (84.6)
	Clinical remission period	52 (15.4)
Symptoms control	Good control	179 (53.1)
	Partial control	151 (44.8)
	Uncontrolled	7 (2.1)
Absence times	0	213 (63.2)
	1 ~ 3	113 (33.5)
	3 ~ 5	11 (3.3)
Whether asthma attack occurred in the past year	No	71 (21.1)
	Yes	266 (78.9)
Relationship with children	father	103 (30.6)
	mother	234 (69.4)
Occupation	farmer	6 (1.8)
	worker	279 (82.8)
	public functionary	20 (5.9)
	freelance occupation	32 (9.5)
Degree of education	Middle school and technical secondary school	82 (24.3)
	Junior college or big book	248 (73.6)
	Postgraduate or above	7 (2.1)
Family structure	father and mother	142 (42.1)
	big family	195 (57.9)
Parenting style	oneself	5 (1.5)
	join your family	332 (98.5)
Mean daily care time (h)	≤ 3	56 (16.6)
	> 3	281 (83.4)
Degree of work affected	no	71 (21.1)
	once in a while	203 (60.2)
	sometimes	63 (18.7)
Home residence	city	281 (83.4)
	village	56 (16.6)
Family monthly income	<¥5,000	7 (2.1)
	¥5000~¥10,000	56 (16.6)
	>¥10,000	274 (81.3)

#### First-aid literacy, coping style, family function and mental health scores of caregivers of asthmatic children

These statistical description are detailed in Table 2.

**Table 2:** Statistical description of all scale score of caregivers (*N* = 337)

Variable	Range of score	$\overline{\mathrm{X}}$ ± SD
First-aid literacy	0 ~ 14	12.28 ± 1.31
CHIP-frequency score	45 ~ 225	199.23 ± 12.31
CHIP-effectiveness score	0 ~ 135	122.10 ± 8.03
FAD	12 ~ 48	28.50 ± 3.90
GHQ-Self-affirmation	0 ~ 9	7.60 ± 1.26
GHQ-Anxiety	0 ~ 5	0.94 ± 1.22
GHQ-Depression	0 ~ 6	0.18 ± 0.51
GHQ-Total score	0 ~ 20	2.53 ± 2.34

Notes: CHIP, Coping Health Inventory for Parents; FAD, Family Assessment device; GHQ, General Mental Health Questionnaire

#### Correlations among the study variables

**Fig. 1:**
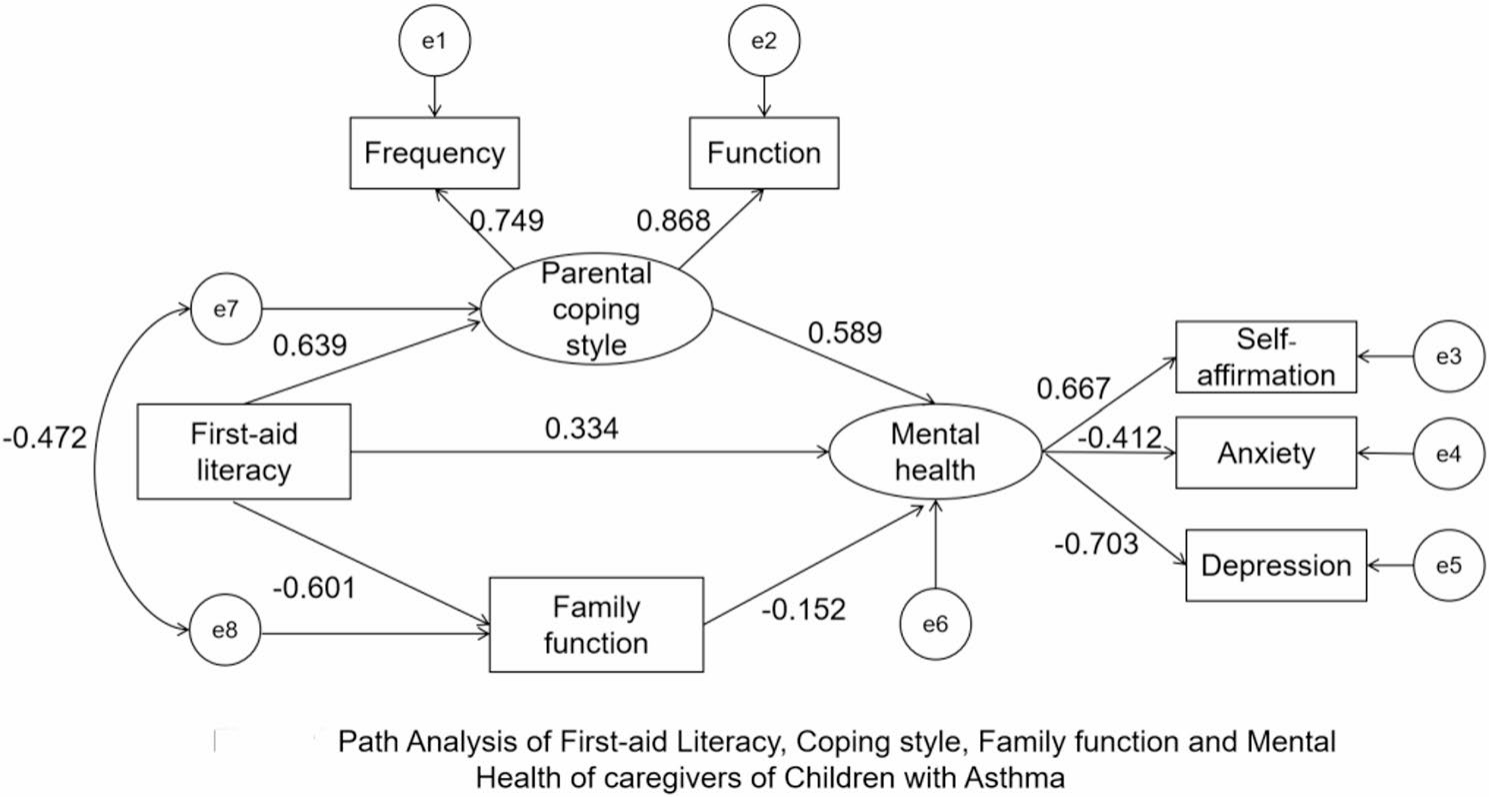
Path analysis of first-aid literacy, coping style, family function and mental health of caregivers of children with asthma

As shown in Table 3, caregivers’ mental health was positively correlated with family functioning, indicating that poorer family functioning was associated with poorer mental health. Mental health was negatively correlated with first-aid literacy and positive coping style, indicating that higher first-aid literacy and more positive coping styles are associated with better caregiver mental health. Based on Pearlin’s caregiver stress model, an SEM for first-aid literacy, coping style, family function, and mental health of caregivers of children with asthma was established. The specific coefficients of action are shown in Fig. 1. The maximum likelihood method was used to fit and modify the model. The model demonstrated a good fit, and all action paths in the model were statistically significant (all *P* < 0.01).

Figure 1 shows that first-aid literacy not only directly impacted caregivers’ mental health but also indirectly impacted it through coping style and family function. To assess the mediating effects, the total, direct, and indirect effects of first-aid literacy on mental health were calculated. The total effect was 0.787 (sum of 0.334 and 0.639 × 0.566 and (-0.601) × (-0.152)), while the direct effect was 0.334. The mediating effect was further tested by the Bootstrap method, and a 95% confidence interval was calculated. The results showed that the indirect effects of first‑aid literacy on mental health via coping style and via family function were both significant (all *P* < 0.01), and the 95% confidence intervals did not include 0, indicating that the mediating effects of coping style and family function were significant. The mediating effect of coping style was 0.362 (0.639 × 0.566), accounting for 46.0% of the total effect. The mediating effect of family functioning was 0.091 (-0.601 ×-0.152), accounting for 11.6% of the total effect.

**Table 3:** Correlation analysis of first-aid literacy, coping style, family functioning and mental health (r)

	First-aid literacy	CHIP-frequency score	CHIP-effectiveness score	FAD	GHQ-Self- affirmation	GHQ-Depression	GHQ-Anxiety	GHQ-Total score
First-aid literacy	1.000							
CHIP-frequency score	0.461^**^	1.000						
CHIP-effectiveness score	0.563^**^	0.650^**^	1.000					
FAD	-0.601^**^	-0.515^**^	-0.580^**^	1.000				
GHQ-Self- affirmation	0.502^**^	0.472^**^	0.514^**^	-0.521^**^	1.000			
GHQ-Depression	-0.296^**^	-0.300^**^	-0.378^**^	0.284^**^	-0.187^**^	1.000		
GHQ-Anxiety	-0.583^**^	-0.427^**^	-0.512^**^	0.496^**^	-0.482^**^	0.332^**^	1.000	
GHQ-Total score	-0.639^**^	-0.542^**^	-0.626^**^	0.601^**^	-0.831^**^	0.490^**^	0.855^**^	1.000

Notes: CHIP, Coping Health Inventory for Parents; FAD, Family Assessment device; GHQ, General Mental Health Questionnaire

First-aid literacy, CHIP-frequency, and CHIP-effectiveness: higher scores = better

Higher scores indicate poorer family functioning

GHQ-Self-affirmation: higher = better; GHQ-Depression & GHQ-Anxiety: higher = worse. GHQ-Total (mental health) = Depression + Anxiety + (reverse of self-affirmation); higher = poorer mental health

**P<0.01

## Discussion

This study contributes to research by introducing Pearlin’s caregiver stress model and SEM to investigate the pathways linking first-aid literacy to caregivers’ mental health in paediatric asthma. Specifically, the study revealed correlations among first-aid literacy, coping style, family function, and mental health, and further demonstrated that first-aid literacy affects mental health both directly and indirectly through coping style and family function. These findings provide a theoretical basis for clinical practice and offer new insights for developing targeted mental health interventions for caregivers of children with asthma.

### Mental health status of caregivers’ of children with asthma

Caregivers’ mental health is a critical yet often overlooked issue in paediatric asthma management. The chronic, relapsing nature of asthma requires caregivers to maintain constant vigilance, make rapid emergency decisions, and adhere to long-term medication plans, all of which can be compromised by psychological distress. Previous studies have reported that 23.56% of caregivers of patients with asthma experience clinically significant anxiety or depression, a prevalence that underscores the urgency of addressing this problem [8]. Moreover, caregivers themselves may not prioritise their own mental health as they tend to focus on their children’s physical symptoms. However, evidence has consistently shown that poor caregiver mental health directly impairs home-based asthma care; anxious or depressed caregivers are more likely to miss medication doses, delay seeking help during exacerbations, and report lower self-efficacy in managing acute attacks [9].

The SEM of our study showed that low mental health (higher GHQ-20 scores) was significantly associated with low first-aid literacy and less positive coping, which in turn weakened family functioning — all essential for safe and effective home care. Notably, the negative correlation between mental health and coping skills reflects the scoring direction of the GHQ-20 (higher scores = poorer mental health); thus, more positive coping was associated with better mental health, consistent with Pearlin’s caregiver stress model. Several factors may explain why our sample showed relatively good mental health despite the caregiving burden. First, participants were predominantly urban residents with higher socioeconomic status and educational attainment, factors associated with lower psychological distress and greater resilience to caregiving stress [40, 41]. Second, public welfare lectures and asthma education provided by the hospital helped caregivers reduce negative emotions stemming from lack of disease-related knowledge [42]. These resources likely contributed to the relatively high levels of self-affirmation observed in our study.

Regarding emotional profiles, we found that anxiety, depression, and self-affirmation coexisted in caregivers, with self-affirmation scores higher than those of anxiety and depression. This aligns with the results of Liu et al. [43] but differs from those of Behmanesh et al. [44]. Although asthma treatment outcomes are generally favourable and many caregivers adapt over time, depressive tendencies can still arise from cumulative stressors such as the child’s frequent school absences, low family income, poor family functioning, and prolonged caregiving burden [45]. Anxiety and depression are distinct but often co-occur. We did not directly compare their severity, but their coexistence with high self-affirmation suggests that positive and negative emotions are not mutually exclusive. The relatively high self-affirmation may be explained by the lengthy diagnostic process of childhood asthma (often nearly one year), during which caregivers gradually accept the child’s illness and adjust their attitudes to maintain a positive state [46]. As a positive emotion, self-affirmation plays an important role in resolving patients’ symptoms and improving psychological outcomes [42]. Related studies have found that increased positive emotions in caregivers promote better quality of care and facilitate children’s recovery [47].

Therefore, although the average GHQ-20 score indicated “good” mental health, this should not be interpreted as absence of risk. Even a modest decline in mental health can affect the caregiving capacity. Clinical nurses should prioritise caregivers’ psychological well-being as a core component of asthma management, actively guide caregivers to maintain self-affirmation, and provide targeted support for those with lower socioeconomic status or limited access to educational resources.

### Relationship between first-aid literacy, coping style, family functioning and mental health

The results showed that the higher the level of functional literacy of caregivers, the lower the care burden and the better their mental health, which is consistent with previous findings [48]. Caregivers can often become anxious and depressed, and paired with lack of accurate cognition and disease-related knowledge, it can lead to confusion and frustration [49]. Researchers have suggested two possible explanations for the significance of first-aid literacy in mental health. First, given that caregivers have to meet the needs of disease management and medication supervision for children along with balancing the relationship between work and family in the process of caring, children’s asthma symptoms can trigger panic, anxiety, or depression if they lack first-aid literacy [50]. Second, improvements first-aid literacy can enhance caregivers’ ability to deal with problems, their work, as well as their own mental health issues.

We also found that caregivers’ coping style was related to their mental health; the more frequently they used positive coping styles, the greater the role of their positive coping style became, and this also improved their mental health, which is consistent with the findings of Fairfax [51]. Researchers have found that coping styles can alleviate depression caused by parenting pressure on caregivers [52]. This is probably because caregivers with high levels of first-aid literacy can effectively adopt positive coping styles and maintain healthy psychological situations. Faced with the long-term stress of disease care, caregivers who adopt a positive coping style tend to have better mental health. Since research has confirmed that depression in caregivers can be effectively reduced by adjusting coping styles for functional disorders [51], medical staff should encourage caregivers to adopt positive coping methods, and improve their ability to cope with the disease and the psychological burden brought about by children’s asthma, when carrying out individualised health education for caregivers.

Furthermore, research has documented that family functioning partially mediates the impact of caregivers’ first-aid literacy on their mental health. Caregivers can maintain harmonious family relationships and healthy family functioning when first-aid literacy is higher. In this study also family functioning showed a positive correlation with caregivers’ mental health, which is consistent with the results of Weinstein [53]. Higher the family functioning level, the better the caregiving performance and the lower the caregiver depression [54]. Conversely, a low level of family functioning leads to disorder in family, aggravating the psychological stress and mental health of caregivers [53]. During the treatment of children with asthma, medical staff should focus on timely communication with caregivers to ensure optimal functioning of the family to enhance their mental well-being.

### Study limitations

This study has several limitations that must be considered. First, all data were collected from a single hospital in Qingdao, Shandong Province, which may limit the generalisability of our findings to families in other regions or healthcare settings. Second, the cross-sectional design precludes causal inferences; therefore, the proposed mediating pathways should be tested in future longitudinal or interventional studies. Third, convenience sampling used in our study may have introduced selection bias and limited representativeness. Fourth, the sample consisted exclusively of parent caregivers; grandparent caregivers in Chinese families who frequently share caregiving responsibilities for children with asthma were excluded. Therefore, future studies should deliberately recruit grandparent caregivers to enable meaningful comparisons between parents and grandparent caregivers, as the two types of caregivers may differ in first-aid literacy, coping style, family function, and mental health outcomes. Additionally, other factors that may influence caregivers‘ mental health were not included in the present SEM model, which is a limitation of this study. Despite these limitations, our findings provide useful directions for future interventions targeting modifiable factors. Specifically, our findings are primarily applicable to similar Chinese urban settings, and may not be directly generalisable to Western or other cultural contexts without further validation. Future cross-cultural studies are required to test the equivalence of this model across different populations.

## Conclusion and implications

This study demonstrated that first-aid literacy directly influenced the mental health of caregivers of children with asthma, and indirectly impacted caregivers’ mental health through two mediating pathways: coping style and family function. These findings indicate that improving first-aid literacy not only directly benefits caregivers’ psychological well-being, but also enhances it by fostering positive coping styles and healthier family functioning. Hence, first-aid training should be incorporated into paediatric asthma care, with additional attention paid to coping styles and family functioning as key mediators. Healthcare providers, particularly nurses, who have frequent contact with caregivers, are well-positioned to deliver first-aid education and psychological support. Improving caregivers’ first-aid literacy through such education can reduce anxiety and enhance their mental health. However, caregivers with clinically significant anxiety or depression should be referred to a mental health specialist for further assessment and treatment.

Improving first-aid literacy through simulation-based training and written action plans may directly enhance caregivers’ mental health. Promoting positive coping styles via cognitive‑behavioural or mindfulness‑based programs could strengthen caregivers‘ resilience to stress. Family centred interventions that improve communication and reduce conflict may optimise family functioning. Given the strong mediating effect of coping styles (46.0% of the total effect), interventions should prioritise coping skills training. Moreover, stepwise care models that first screen for psychological distress and then provide tailored support (from brief education to referral for clinical treatment) can allocate resources efficiently. Future randomised controlled trials are needed to evaluate the effectiveness of such multicomponent interventions for caregivers of patients with asthma.

## Supplementary Information

Below is the link to the electronic supplementary material.

Supplementary Material 1

## Data Availability

The data that support the findings of this study are available from the corresponding author upon reasonable request.

## Abbreviations

SEM: Structural equation model
CFI: Comparative fit index
RMSEA: Root mean square error of approximation
SRMR: Standardized root mean square residual
