# Supplementary material for: Relationship between first-aid literacy, coping style, family functioning and mental health of caregivers of children with asthma
Source: BMC Nurs. 2026 May 31;25:685. doi: 10.1186/s12912-026-04829-z (PMC13439885; doi:10.1186/s12912-026-04829-z)
Supplement: Supplementary file 1 [file 12912_2026_4829_MOESM1_ESM.doc]

****The General Data Questionnaire****

Hello ! Thank you very much for participating in this study ! We wish your child a speedy recovery! Please tick “✓” in the corresponding option or fill in the relevant items.

****Children with Asthma:****

1. Gender: □ Male □ Female
2. Age: ______ years
3. Only child: □ Yes □ No
4. Child’s education level: □ At home □ Kindergarten □ Preschool □ Primary school
5. Duration of illness:
    □ Less than 1 year □ 1-2 years □ 3-5 years □ More than 5 years
6. Number of medical visits in the past year:
    □ 0 times □ 1-3 times □ 3-5 times □ More than 5 times
7. Number of school absences in the past year:
    □ 0 times □ 1-3 times □ 3-5 times □ More than 5 times
8. Number of hospitalizations in the past year:
    □ 0 times □ 1-3 times □ 3-5 times □ More than 5 times
9. Number of asthma attacks in the past year:
    □ None □ 1-3 times □ 3-5 times □ More than 5 times
10. Cost of asthma treatment in the past year:
     □ <1000 RMB □ 1000-5000 RMB □ 5000-10000 RMB □ >10000 RMB
11. Family history of asthma: □ Yes □ No
12. Medical payment method:
     □ Social health insurance □ Rural cooperative medical system □ Self-funded

□ Other

****Caregivers of the Child:****
13. Gender: □ Male □ Female
14. Age: ______ years
15. Relationship to the child:

□ Father □ Mother □ Grandparents (paternal) □ Grandparents (maternal)
16. Occupation: □ Farmer □ Worker □ Civil servant □ Self-employed
17. Education level:
 □ Primary school or below □ Junior high school □ High school or technical secondary school □ Associate or Bachelor’s degree □ Master’s degree or above
18. Family structure:

□ Single-parent □ Two-parent □ Extended family (three generations living together) □ Reconstituted family
19. Child-rearing method: □ By oneself □ Together with family members
20. Family relationship: □ Harmonious □ Fair □ Poor □ Separated
21. Average daily care time: □ ≤3 hours □ >3 hours
22. Impact on work: □ None □ Occasionally □ Sometimes □ Often □ Always
23. Family location: □ Urban □ Suburban □ Rural
24. Monthly household income:
 □ ≤3000 RMB □ 3000-5000 RMB □ 5000-10000 RMB □ >10000 RMB
25. Monthly per capita household income:
 □ ≤1000 RMB □ 1000-3000 RMB □ 3000-5000 RMB □ >5000 RMB

****The following content is to be completed by the researchers:****
26. Disease Stage:
 □ Chronic Persistent Stage: In the past 3 months, symptoms such as wheezing, coughing, shortness of breath, and chest tightness have occurred with varying frequency and/or intensity.
 □ Clinical Remission Stage: Symptoms and signs have disappeared after treatment or without treatment, and lung function levels have returned to pre-acute exacerbation levels, maintained for more than 3 months.
27. Symptom Control Level: Please answer based on the actual situation in the last 4 weeks.
① For children < 6 years old:
a. Daytime symptoms lasting at least a few minutes occur more than once a week.
b. Waking up at night due to asthma or coughing.
c. Use of rescue reliever medication more than once a week.
d. Activity limitation due to asthma (runs/plays less than other children, tires easily when walking/playing).
② For children ≥ 6 years old:
a. Daytime symptoms ______ times/week.
b. Waking up at night due to asthma.
c. Use of rescue reliever medication more than twice a week.
d. Activity limitation due to asthma.
□ Well controlled: None of the above symptoms are present.
□ Partly controlled: 1-2 of the above symptoms are present.
□ Uncontrolled: 3-4 of the above symptoms are present.
